# Exploring the natural products chemical space through a molecular search to discover potential inhibitors that target the hypoxia-inducible factor (HIF) prolyl hydroxylase domain (PHD)

**DOI:** 10.3389/fphar.2023.1202128

**Published:** 2023-08-21

**Authors:** Abrar Mohammad Sayaf, Saif Ullah Khalid, Jawad Ahmed Hameed, Abdulrahman Alshammari, Abbas Khan, Anwar Mohammad, Saeed Alghamdi, Dong-Qing Wei, KarKheng Yeoh

**Affiliations:** ^1^ School of Chemical Sciences, Universiti Sains Malaysia, Gelugor, Penang, Malaysia; ^2^ Islamia University of Bahawalpur, Bahawalpur, Punjab, Pakistan; ^3^ Nawaz Sharif Medical College, Jalalpur Jattan, Gujrat, Punjab, Pakistan; ^4^ Department of Pharmacology and Toxicology, College of Pharmacy, King Saud University, Riyadh, Saudi Arabia; ^5^ Department of Bioinformatics and Biological Statistics, School of Life Sciences and Biotechnology, Shanghai Jiao Tong University, Shanghai, China; ^6^ Zhongjing Research and Industrialization Institute of Chinese Medicine, Nayang, Henan, China; ^7^ Department of Biochemistry and Molecular Biology, Dasman Diabetes Institute, Dasman, Kuwait; ^8^ Department of Pharmacy, Riyadh Security Forces Hospital, Ministry of Interior, Riyadh, Saudi Arabia; ^9^ State Key Laboratory of Microbial Metabolism, Joint Laboratory of International Laboratory of Metabolic and Developmental Sciences, Shanghai-Islamabad-Belgrade Joint Innovation Center on Antibacterial Resistances, Ministry of Education and School of Life Sciences and Biotechnology, Shanghai Jiao Tong University, Shanghai, China; ^10^ Peng Cheng Laboratory, Shenzhen, Guangdong, China

**Keywords:** HIF, molecular screening, natural products, molecular simulation, binding free energy

## Abstract

**Introduction:** Hypoxia-inducible factor (HIF) prolyl hydroxylase domain (PHD) enzymes are major therapeutic targets of anemia and ischemic/hypoxia diseases. To overcome safety issues, liver failure, and problems associated with on-/off-targets, natural products due to their novel and unique structures offer promising alternatives as drug targets.

**Methods:** In the current study, the Marine Natural Products, North African, South African, East African, and North-East African chemical space was explored for HIF-PHD inhibitors discovery through molecular search, and the final hits were validated using molecular simulation and free energy calculation approaches.

**Results:** Our results revealed that CMNPD13808 with a docking score of −8.690 kcal/mol, CID15081178 with a docking score of −8.027 kcal/mol, CID71496944 with a docking score of −8.48 kcal/mol and CID11821407 with a docking score of −7.78 kcal/mol possess stronger activity than the control N-[(4-hydroxy-8-iodoisoquinolin-3-yl)carbonyl]glycine, 4HG (−6.87 kcal/mol). Interaction analysis revealed that the target compounds interact with Gln239, Tyr310, Tyr329, Arg383 and Trp389 residues, and chelate the active site iron in a bidentate manner in PHD2. Molecular simulation revealed that these target hits robustly block the PHD2 active site by demonstrating stable dynamics. Furthermore, the half-life of the Arg383 hydrogen bond with the target ligands, which is an important factor for PHD2 inhibition, remained almost constant in all the complexes during the simulation. Finally, the total binding free energy of each complex was calculated as CMNPD13808-PHD2 −72.91 kcal/mol, CID15081178-PHD2 −65.55 kcal/mol, CID71496944-PHD2 −68.47 kcal/mol, and CID11821407-PHD2 −62.06 kcal/mol, respectively.

**Conclusion:** The results show the compounds possess good activity in contrast to the control drug (4HG) and need further *in vitro* and *in vivo* validation for possible usage as potential drugs against HIF-PHD2-associated diseases.

## 1 Introduction

Hypoxia-inducible factor (HIF) is a heterodimeric transcription factor that is made up of two helix-loop-helix proteins, HIF-α and HIF-β. HIF is a prime controller for cellular oxygen homeostasis, whereas the prolyl hydroxylase domain (PHD) enzyme is a vital regulator for HIF-α stability (Oh et al., 2015). Promoting the stability and function of HIF through PHD inhibition has been recommended as a potent medicinal approach for ischemic diseases, cancer-related fatigue, and other oxidative pathological disorders resulting from anemia (Wu et al., 2016; Wu et al., 2017). Under normoxia, HIF-α has a comparatively short half-life (less than 5 min) and it quickly decomposes through the ubiquitin-proteasome pathway. Both proline residues of human HIF-1α, Pro402, and Pro564, within its oxygen-degradation domain (ODD), undergo oxygen-dependent prolyl hydroxylation catalyzed by prolyl hydroxylase domain enzymes (PHD1, 2 and 3) in the presence of 2-oxoglutarate (2OG) and Fe^2+^ ion (Chowdhury, 2017). Prolyl hydroxylation of HIF-α then enhances its linking to the tumor suppressor protein called von Hippel-Lindau (pVHL), which is a fragment of the E3-ubiquitin composite, causing proteasomal degradation of HIF-α. On the other hand, *ß*-hydroxylation of HIF-1α at its asparagine residue (Asn803) within the C-terminal transcriptional activation domain (C-TAD) is catalyzed by factor inhibiting HIF (FIH). This modification lowers HIF-α bonding to coactivator p300-CBP proteins, rendering the inactivation of HIF transcriptional action (Hampton-Smith et al., 2019).

Under conditions of hypoxia, reduced oxygen levels inhibit the activity of PHDs, thus preventing HIF- α prolyl hydroxylation and its degradation. Then, accumulated HIF-α is translocated into the nucleus and forms heterodimer by binding to HIF-β, this subsequently leads to upregulation of HIF transcriptional pathways (He et al., 2012). Recent studies revealed PHD2 is a significant oxygen sensor and a major HIF-α regulator, so considering its potency as a therapeutic target, most inhibitors were designed (Emmanuel et al., 2003; Ashok et al., 2017). Many of the reported PHD2 inhibitors compete with 2OG co-substrate to bind to its active site (Rosen et al., 2010; Pergola et al., 2016).

Many PHD drug candidates have been investigated but some side effects and limitations such as liver failure and hypertension were also reported (Del Vecchio and Locatelli, 2018). Natural products which have novel and unique structures have been a principal source of drugs for many illnesses [11–13]. Exploration of the natural product chemical space provides an alternative way for the discovery of novel PHD drug candidates. The objectives of the current study are to discover novel potential inhibitors from Comprehensive Marine Natural Products (CMNPD), North African, East African, North-East African, and South African Natural Products databases to target PHD2 protein. The co-crystal structure of HIF-PHD2 was retrieved from a protein databank in a complex with N-[(4-hydroxy-8-iodoisoquinolin-3-yl)carbonyl]glycine (4HG). In the current study, 4HG was used as a positive control and the best compounds were subjected to molecular simulation and binding free energy calculations. Our potential shortlisted candidates that demonstrated promising activity against PHD2 need further *in vitro* and *in vivo* validation for the development of potential drugs against HIF-associated diseases.

## 2 Methodology

### 2.1 Description of methodology

For docking studies, the complex crystal structure of 4HG ligand and HIF-PHD2 (PDB ID:2G19) was obtained from Research Collaboratory for Structural Bioinformatics, Protein Data Bank (RCSB, PDB) (Rose et al., 2016). The complex structure of PHD2-4HG was then subjected to PyMOL for the removal of water molecules while retaining the inhibitor and Fe^2+^ ion in the receptor. The addition of hydrogen atoms and minimization of the protein structure was achieved by using Chimera (Pettersen et al., 2004; Goddard et al., 2005).

### 2.2 Data set preparation

The 3D-SDF format of African natural products from the North, South, East, and North-East African regions were downloaded from the African Natural Products Database (ANPDB) and the South African Natural Compounds Database (SANCDB) websites (http://african-compounds.org/anpdb/) (https://sancdb.rubi.ru.ac.za/). These databases consist of compounds with varied therapeutic significance (Ntie-Kang et al., 2013; Lyu et al., 2021). Then the FAF-Drugs4 (https://fafdrugs4.rpbs.univ-paris-diderot.fr/) web server was used to filter only those molecules which obey Lipinski’s rule of five (Lagorce et al., 2017). The approved molecules by the Lipinski rule were then prepared and subjected to screening (Yi et al., 2019). The prepared and filtered natural products were then screened against the active pocket of PHD2.

### 2.3 Positive control docking

The extracted 4HG was re-docked into the PHD2 cavity to validate the effectiveness of the docking procedures. Ligand docking tasks in EasyDock Vina 2.0 were used to perform the docking. Before virtual screening of the positive control, the active site residues Tyr310, His313, Asp315, Tyr329, His374, and Arg383 of protein were selected to create the grid with dimensions of X = 4.76, Y = 46.87, and Z = 24.4.

### 2.4 Virtual drugs screening

For the virtual screening of all databases, EasyDock Vina 2.0 was used. The AutoDock4 algorithm was used by EasyDock Vina to screen and rank all the best drug-like natural products. Before the screening, all molecules were converted to PDBQT format. The high-scored compounds were then nominated for screening at 64 exhaustiveness (Teli et al., 2021). To eliminate false-positive outcomes, a second time screening was conducted to re-evaluate the ranked compounds. Then, for the top 10% of drugs selected from each database, induced fit docking (IFD), which supports the flexibility of receptors and ligands, was conducted (Ravindranath et al., 2015). This docking methodology also uses similar modes like EasyDock Vina 2.0 but is less time-consuming and precise. Finally, to validate the final best-hit compounds further, analysis such as molecular dynamics simulation and free energy calculation methods were used.

### 2.5 Ligands-protein visualization

To observe the protein-ligand interactions Schrödinger Maestro free Academic version 2018–1 (for visualization only) and PyMOL software were used (Yuan et al., 2017; Maestro, 2020).

### 2.6 Molecular dynamics simulation

Atomistic investigation of the binding of the top hits to the PHD2 active pocket was achieved by using the AMBER21 simulation tool (Case et al., 2005). The antechamber, an integrated module in AMBER, was used to generate the drug topologies while the Amber general force field (GAFF) and ff19SB force fields were recruited for the solvated complexes to complete the simulation. An optimal point charge (OPC) solvation model and sodium (Na^+^) ions were added for neutralization. Multi-step energy minimization followed by heating and equilibration was completed to relax the structure and remove discrepancies in the complexes. The Particle Mesh Ewald (PME) algorithm (Price and Brooks III 2004), was used to treat long-range electrostatic interactions, Van der Waals interactions, and short-range Columbic interactions were used, while for Langevin thermostat and Berendsen barostat were used for temperature and pressure control. A 100 ns simulation for each complex with a time step of 2fs was performed. The dynamics, stability, and other features of the ligand-protein complexes were evaluated by using CPPTRAJ and PTRAJ (Roe and Cheatham, 2013). Root mean square deviation (RMSD) was used for dynamic stability, root mean square fluctuation (RMSF)for residues flexibility indexing, radius of gyration (Rg) for protein size measurement, and hydrogen bonding in the trajectories were estimated for interaction landscape.

### 2.7 Binding free energy calculation using MM/GBSA

The MM/GBSA technique is frequently employed in the field of drug development. This approach can be used to determine the most promising candidates, by predicting the essential interactions, and improve the quality of a lead molecule and specificity by computing the binding free energy of a ligand and a protein. This approach combines molecular mechanics simulations, which describe the interactions between atoms, with implicit solvent models, which describe the interactions between the protein and solvent (Li et al., 2011; Khan et al., 2020; Khan et al., 2021; Teli et al., 2021; Khan et al., 2022a). Hence, we applied this approach here to accurately compute the total binding free energy of the protein-ligand complexes. The binding free energy can be estimated mathematically as:

$$\prime \prime \Delta G\left(bind\right)=\Delta G\left(complex\right)-\left[\Delta G\left(receptor\right)+\Delta G\left(ligand\right)\right]\prime \prime$$ (1)

Different contributing components of total binding energy were calculated by the following equation:

″G=Gbond+Gele+GvdW+Gpol+Gnpol″ (2)

It has a wide range of applications. For example, the equation has been used to estimate the binding energy for protein-protein or protein-ligand complexes in different studies including SARS-CoV-2 and neurological disorders (Fu et al., 2018; Xue et al., 2018; Arshia et al., 2021; Jomhori et al., 2021; Fu et al., 2022; Xue et al., 2022).

## 3 Results and discussion

**FIGURE 1:**
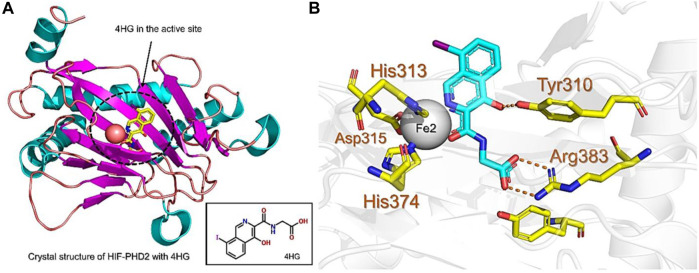
**(A)** Stereoview ribbons representation of the secondary structure of the catalytic domain of PHD2 complexed with N-[(4-hydroxy-8-iodoisoquinolin-3-yl)carbonyl]glycine, 4HG (yellow) and the active site Fe^2+^ (orange sphere) (PDB ID: 2G19). The structure of 4HG is shown in the small box. **(B)** The interaction of 4HG with the active site residues of PHD2. Arg383 is one of the most essential residues in the functionality of PHD2.

PHD enzymes play an important role in oxygen sensing and HIF regulation and have been associated with various essential human disorders such as anemia, inflammation, cancer, rheumatoid arthritis, strokes, spinal cord injury, and von Hippel–Lindau disease-related renal cell carcinoma (Kaelin Jr, 2004; Semenza, 2014; Kiriakidis et al., 2017). In this study, we explored several natural products’ chemical spaces for the discovery of novel PHD inhibitors. The co-crystal structure of PHD2 in complex with N-[(4-hydroxy-8-iodoisoquinolin-3-yl)carbonyl]glycine (4HG) (Figure 1A) was first retrieved from the protein data bank. For the identification of potential PHD inhibitors, CMNPD (comprehensive marine natural products database, and African Natural Products databases were screened against the PHD2 active site. Potential drug candidates were compared with the positive control, 4HG, which had a docking score of −6.87 kcal/mol. The crystal structure reveals that the nitrogen atom in the aromatic ring and the amide carbonyl oxygen of 4HG chelate the active site iron in a bidentate manner. The carboxylic acid in the glycinamide group occupies the 2OG binding pocket and is positioned to form a salt bridge with Arg383, and the 3-hydroxyl group in the isoquinoline moiety hydrogen bonds with the nearby Tyr310 residue in the PHD2 active site as shown in Figure 1B. Thus, in this study, we searched for potential natural products that can chelate and bind strongly to PHD2 active site by interacting with these key residues. We discovered four target-hit compounds from screening a total of 46,318 compounds from the CMNPD and African Natural Products databases. The interaction and binding energies of these shortlisted top hits are better than the co-crystalized structures reported with other drugs.

### 3.1 Exploring the comprehensive marine natural products chemical space

**FIGURE 2:**
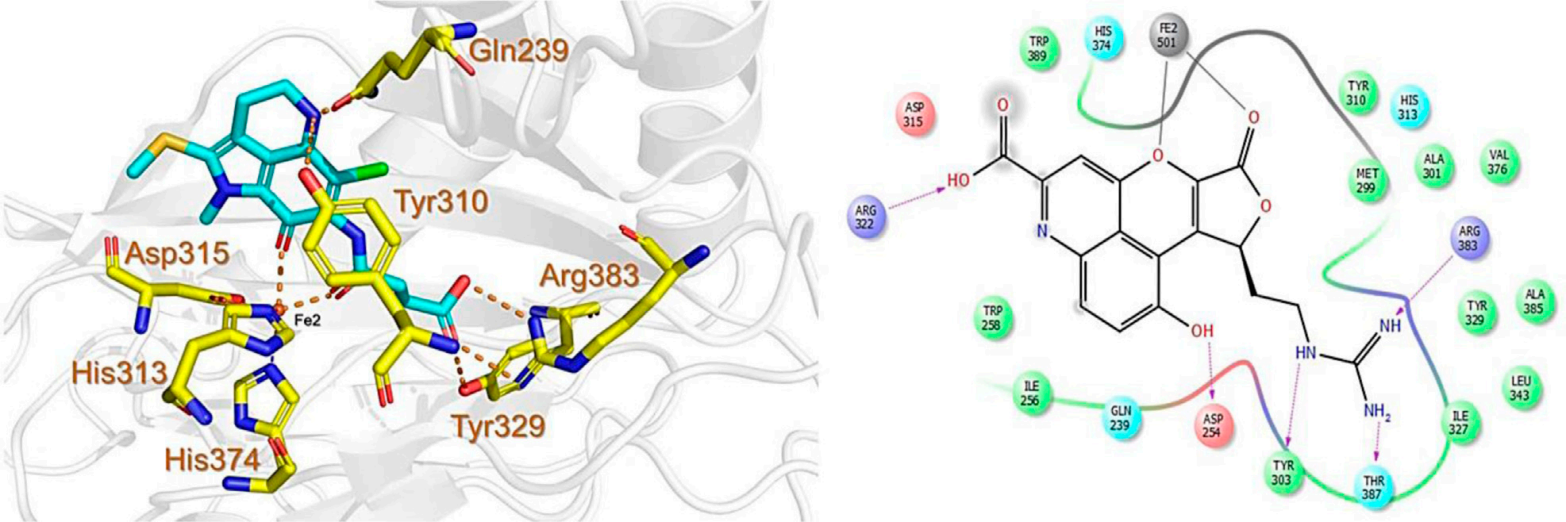
Binding mode of Distomadine B (CMNPD13808) in the active site of PHD2. The left panel shows the 3D interaction pattern while the right panel shows the 2D interaction pattern. (Schrödinger Maestro academic version for visualization only).

The CMNPD has over 32,000 compounds derived from 3,400 organisms. The pre-filter of 32,000 compounds from the CMNPD using Lipinski’s rule of five (R5′) revealed 23,764 compounds that obey R5′ while the rest were discarded. The initial screening revealed 407 compounds that had better docking scores than the control drug 4HG, ranging from −7.03 kcal/mol to −8.69 kcal/mol. The top 10% of the compounds (41 compounds) were re-evaluated for interactions with the PHD2 active site residues. Induced fit docking (IFD) subsequently revealed docking scores of −7.36 kcal/mol to −8.69 kcal/mol. The biologically active compound, Distomadine B from *Pseudodistoma aureum* with compound ID CMNPD13808, is a tricyclic guanidine-containing 6-hydroxyquinoline alkaloid (Michael, 2007). CMNPD13808 was found to have the highest docking score, i.e., −8.69 kcal/mol, among the screened compounds and was later selected for interaction evaluation and molecular simulation studies. Considering the essential residues that are required for the function of PHD2, CMNPD13808 may target and block the 2OG binding pocket more potently than the control ligand 4HG. The 3D and 2D interaction patterns of Distomadine B (CMNPD13808) in the PHD2 active site are shown in Figure 2. The oxygen of its furanone carbonyl group and pyran ring chelate the active site iron in a bidentate manner, while the carboxyl and hydroxyl group in its quinolone ring form hydrogen bonds with Arg322 and Asp254. Moreover, its guanidine side chain was also capable of forming hydrogen bonds with Thr387, Tyr303, and Arg383. Molecular docking studies indicate CMNPD13808 may robustly block the PHD2 2OG binding pocket, therefore, making it a potential PHD2 drug candidate.

### 3.2 Exploring the African natural products chemical space

**FIGURE 3:**
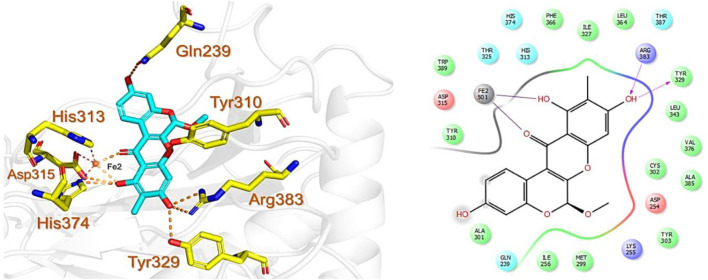
Binding mode of Boeravinone D (CID15081178) in PHD2 binding pocket. The left panel shows the 3D interaction pattern while the right panel shows the 2D interaction pattern. (Schrödinger Maestro academic version for visualization only).

Similarly, the North, East, North-East, and South African Natural Products databases with a total of 14,318 compounds were screened for potential active compounds against PHD2 using the double screening protocol. Following the same criteria, the four databases from African geographical regions were pre-filtered using R5’. The top three compounds with the highest docking scores were selected for interaction and simulation analysis. The second hit compound identified from SANCDB was 3,9,11-trihydroxy-6-methoxy-10-methyl-6H-chromeno[3,4-b]chromen-12-one or Boeravinone D (CID15081178), which gave a docking score of −8.027 kcal/mol. Boeravinone D was originally isolated from *Abronia nana* and has previously been reported to have potent anti-oxidative, genoprotective, spasmolytic, and anti-breast cancer effects (Park et al., 2014). Figure 3 shows the binding mode of CID15081178 in the PHD2 binding pocket. CID15081178 chelates the active site Fe^2+^ with its carbonyl and 11-hydroxyl oxygen while its 9-hydroxyl group interacts with Tyr329 and Arg383 via hydrogen bonding.

**FIGURE 4:**
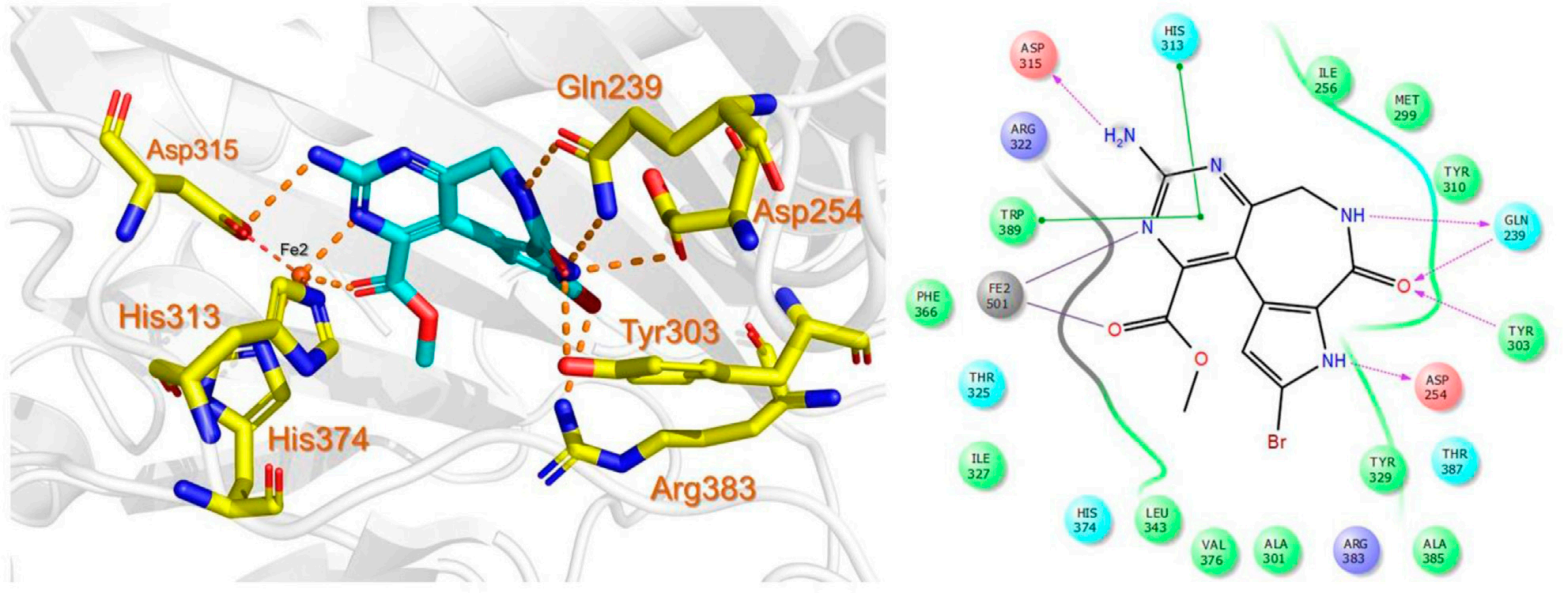
Binding mode of 3-debromolatonduine B methyl ester (CID71496944) in the active site of PHD2. The left panel shows the 3D interaction pattern while the right panel shows the 2D interaction pattern.

The bioactive alkaloid, 3-debromolatonduine B methyl ester (CID71496944) isolated from marine sponge *Stylissa species*, with the docking score of −8.48 kcal/mol, was the third hit compound identified from the East African Natural Products database (EANPDB) (Fouad et al., 2012, !!! INVALID CITATION !!! {}). The binding mode of CID71496944 in the PHD2 active site is given in Figure 4. CID71496944 chelates the active site iron with its pyrimidine nitrogen and the carboxyl oxygen in a bidentate fashion. Interestingly, its amino group forms hydrogen bonds with Asp315 while its pyrimidine ring interacts with His313 and Trp389 via π-π stacking. Furthermore, the amino group and carbonyl oxygen in its azepine-2-one ring and the amino group in its pyrrole ring could also form hydrogen bonding with Gln239, Tyr303, and Asp254, respectively. Interestingly, the docking results show CID71496944 does not form strong interaction with Arg383, which is consistent with the binding properties observed in Molidustat, 2-[(1,10-Biphenyl)-4-yl]-8-[(1-methyl-1H-imidazol-2-yl)methyl]-2,8 diazaspiro [4.5] decan-1-one, 6-[5-hydroxy-4-(1H-1,2,3-triazol-1-yl)-1H-pyrazol-1-yl]nicotinicacid, and spiro [4.5] decanones PHD2 inhibitors (McDonough et al., 2005; Chowdhury et al., 2009; Deng et al., 2013; Holt-Martyn t al. 2019).

**FIGURE 5:**
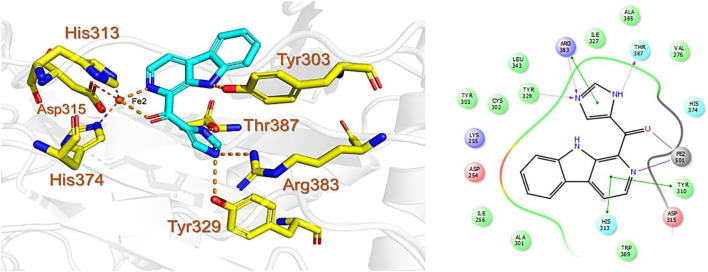
Binding mode of Des-N-Methylxestomanzamine A (CID11821407) in the active pocket of PHD2. The left panel shows the 3D interaction pattern while the right panel shows the 2D interaction pattern. (Schrödinger Maestro academic version for visualization only).

The last target hit compound was Des-N-methylxestomanzamine A (CID11821407) with a docking score of −7.78 kcal/mol. CID11821407 is a manzamine alkaloid that was identified from both CMNPD and EANPDB in our screening. CID11821407 has previously been reported to have strong activity against infectious and tropical parasitic diseases (Rao et al., 2003). The interaction pattern of CID11821407 with PHD2 is given in Figure 5. The pyridine nitrogen atom and the carbonyl oxygen atom are positioned to coordinate in a bidentate manner with the active site iron. The pyridine ring interacts with Tyr310 and His313 via π-π stacking, while the nitrogen and amino groups in its imidazole ring interact with Arg383 and Tyr329, and Thr387 via hydrogen bonding. In addition, the imidazole ring could also interact with Arg383 through π-π stacking. Overall, the current findings demonstrate that the shortlisted top-hit compounds with higher docking scores may inhibit PHD2 more potently than the control molecule 4HG. Therefore, validation was performed using molecular simulation and free energy calculation was subsequently carried out. The 2D structures of the top hit compounds and their docking scores were summarized in Table 1.

**TABLE 1:** 2D structures of the top hits and control ligands with their names, database IDs, docking scores, interacting residues, and nature are given.

S. No	2D structure	Names	Database ID	Docking score	Interactions	Interaction Nature
1	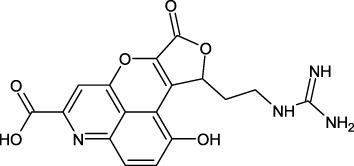	Distomadine B	CMNPD13808	−8.690	Fe^2+^	Metals co-ordination
					Tyr310	H-bond
					Thr387	H-bond
					Arg 322 and Arg383	H-bond
						H-bond
2	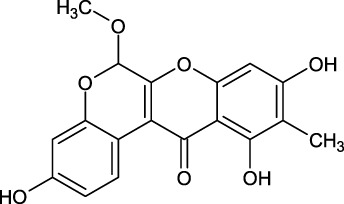	Boeravinone D (3,9,11-trihydroxy-6-methoxy-10-methyl-6H-chromeno[3,4-b]chromen-12-one)	CID15081178	−8.027	Fe^2+^	Metal co-ordination
					Tyr329 and Arg383	H-bond
						H-bond
3	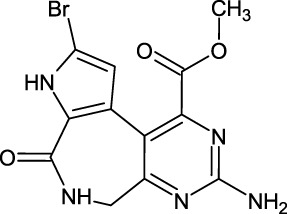	3-Debromolatonduine B methyl ester	CID71496944	−8.48	Fe^2+^	Metals co-ordination
					Gln239, Tyr303, His313	H-bond
					Trp 389 Asp315 and Asp254	H-bond
						Pi-Pi
						Pi-Pi
						H-bond
						H-bond
4	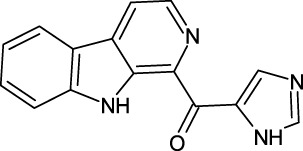	Des-N-Methylxestomanzamine A	CID11821407	−7.78	Fe^2+^	Metal co-ordination
					Tyr310, His313, Tyr329, Thr387 and Arg383	Pi-Pi
						Pi-Pi
						H-bond
						H-bond
						H-bond, Pi-Pi
5	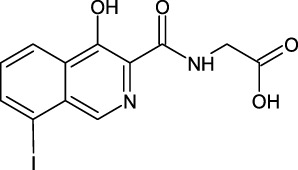	(1-chloro-4-hydroxyisoquinoline-3-carbonyl)glycine	4HG	−6.87	Fe^2+^	Metals co-ordination
					Tyr310, Tyr329 and Arg383	H-bond
						H-bond
						H-bond

### 3.3 Dynamic stability analysis of the top hits

**FIGURE 6:**
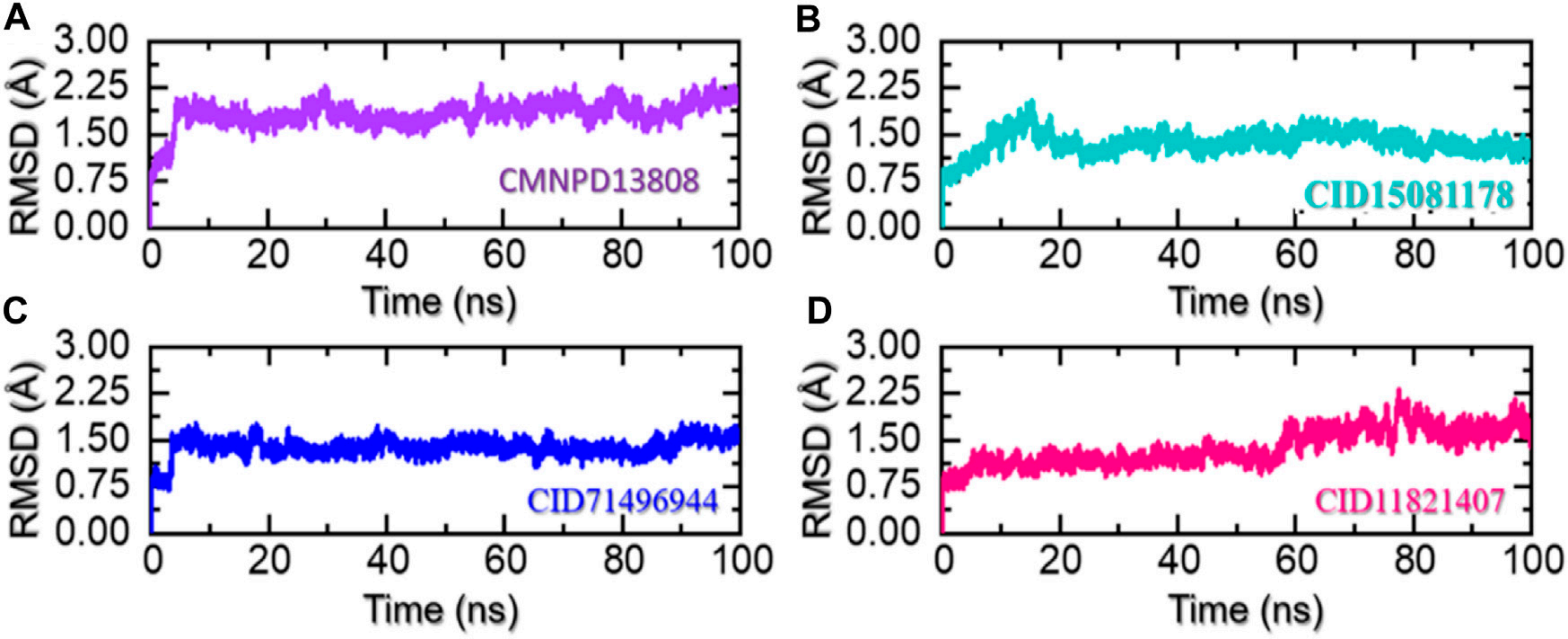
Stability of the ligand-bound protein complexes in solvated model. **(A)** Demonstrate the RMSD for CMNPD13808-PHD2. **(B)** Demonstrate the RMSD for CID15081178-PHD2. **(C)** Demonstrate the RMSD for CID71496944-PHD2. **(D)** Demonstrate the RMSD for CID11821407-PHD2.

In molecular dynamics simulation and structural biology, root-mean-square deviation (RMSD) is often used as a measure of the differences and to evaluate the accuracy of the structure of a protein. It can be used to identify the areas of a protein structure that are significantly affected by drug binding or other factors and provide insight into the dynamics of protein interactions (Sargsyan et al., 2017). RMSD is used to assess the stability of a protein over time and monitor changes in its structure. By comparing the RMSD values of a protein structure at different stages of a simulation, essential information can be obtained that causes changes in proteins in response to various conditions and make predictions about its behavior in real-world environments. Considering the higher importance of this approach in scaling the dynamic stability of a ligand-bound protein complex, we also used simulation trajectories to calculate the RMSD of each complex. As given in Figure 6A, the complex CMNPD13808-PHD2 demonstrated stable dynamic behavior. Initially, the RMSD increased during the first 10ns and an abrupt increase at 5 ns was reported for this complex. Then afterward the structure RMSD stabilized at 2.0 Å soon after reaching 8 ns With a uniform pattern, the complex reported an average RMSD of 1.96 Å which demonstrates a stable complex during the simulation. No significant perturbation was observed except a minor up and down between 70 and 80 ns. The RMSD overall demonstrates a stable behavior thus showing the imposed pharmacological behavior of the drug in the binding cavity. In the case of the CID15081178 -PHD2 complex, the RMSD was observed to be lower in contrast to the CMNPD13808-PHD2 complex. From the very beginning, an incline in the RMSD graph was observed, however, soon after reaching 1.75 Å at 15 ns the RMSD abruptly declined and continued to follow a stable uniform behavior with no significant perturbation. An average RMSD for this complex was calculated to be 1.45 Å which also implies the pharmacological potential of the drug in blocking the PHD2 active site. The RMSD pattern of the CID15081178 -PHD2 complex is given in Figure 6B. Furthermore, the CID71496944-PHD2 complex also demonstrated stable dynamics and showed similar pharmacological features by demonstrating stable dynamics when bound to the PHD2 active pocket. The RMSD reported an abrupt increase at the beginning and then stabilized at 1.5 Å with no significant dynamic structural perturbation until the end of the simulation. With minor deviations at 40, 65, and 88 ns this complex demonstrated favorable dynamic behavior till 200 ns An average RMSD was calculated to be 1.48 Å. The RMSD pattern of the CID71496944-PHD2 complex is given in Figure 6C. Despite the stable dynamics at the start of the simulation until 58 ns, a continuous increase in the RMSD pattern was observed for the rest of the simulation for the CID11821407-PHD2 complex. As shown in Figure 6D, despite the increase in the RMSD in the later part of the simulation, an average RMSD for this complex was calculated to be in an acceptable range of 1.49 Å. Overall, the results strongly imply how these top-hit ligands strongly bind to the active pockets residues steered by hydrogen bonds and other contacts that are preserved during the simulation and instigate the pharmacological action by blocking the essential residues required for PHD2 functionality. These stable behaviors demonstrate pharmacological potential as the formation of stable complexes by these top-hit molecules may produce the desired therapeutic effects. Furthermore, all the complexes converged with each other, thus showing that they attained a similar atomic configuration during the simulation and satisfy the accuracy of the selected molecules. Consequently, these results may contribute to the development of new treatments for a wide range of diseases, including anemia, inflammation, cancer, rheumatoid arthritis, strokes, spinal cord injury, and von Hippel–Lindau disease-associated renal cell carcinoma mediated by HIF-PHD2.

### 3.4 Protein structural packing through the radius of gyration (Rg) estimation

**FIGURE 7:**
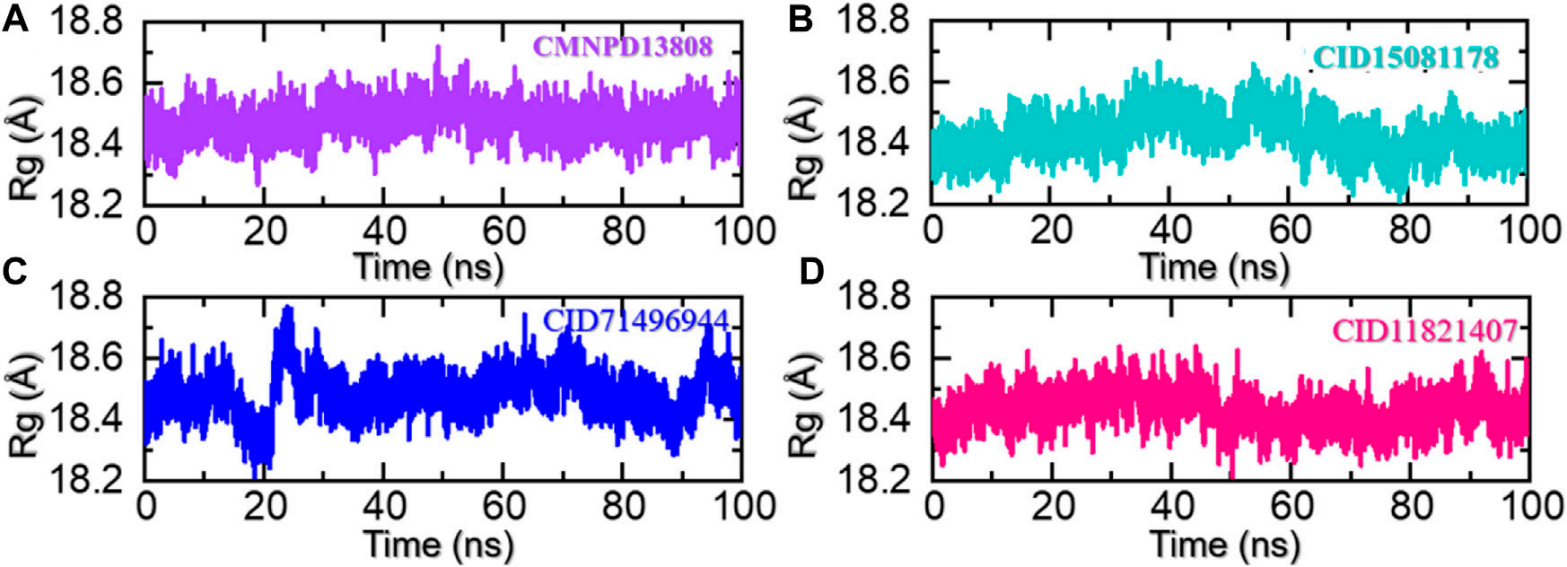
Rg analysis of the ligand-bound protein complexes in the solvated model. **(A)** The Rg for CMNPD13808-PHD2 is demonstrated. **(B)** The Rg for CID15081178 -PHD2 is demonstrated. **(C)** The Rg for CID71496944-PHD2 is demonstrated. **(D)** The Rg for CID11821407-PHD2 is demonstrated.

The radius of gyration (Rg) is a measure of the spatial distribution of the mass of a protein molecule, which determines the size and shape of a protein and can provide important information about its stability and behavior during molecular dynamics simulations. The radius of gyration of a macromolecule can change as the protein interacts with other molecules such as another protein, peptide, or small molecules and as its conformation changes over time. Changes in the Rg can provide insights into the structural dynamics of a protein, including its folding/unfolding, binding/unbinding, and the formation of specific interactions with other molecules. In addition, it also provides information regarding the regions of a receptor that are either flexible or rigid (Arnittali et al., 2019). This information can be used to design new drugs or to predict the behavior of proteins in different environments. To uncover the underlying mechanisms of the top hits in this study that govern their behavior during simulations we also calculated Rg as a function of time. As given in Figures 7A–D, all the complexes demonstrated a similar range of Rg values. For the CMNPD13808-PHD2 complex, the Rg did not report any major perturbation with a minor increment between 40 and 60 ns and then smaller deviations at different time intervals. Moreover, the average Rg for the CMNPD13808-PHD2 complex was calculated to be 18.55 Å and is presented in Figure 7A. Similarly, the CID15081178 -PHD2 complex had similar behavior as its RMSD demonstrated a wave-like pattern in the Rg graph with no significant dynamic structural perturbation, showing minimal unbinding events during the simulation. An average Rg for the CID15081178 -PHD2 complex was estimated to be 18.35 Å. The Rg graph for the CID15081178 -PHD2 complex is given in Figure 7B. Interestingly the CID71496944-PHD2 complex showed similar behavior as its RMSD by demonstrating an abrupt increase/decrease in the Rg initially and then a uniform pattern was observed till the end of the simulation. This shows consistent dynamic behavior with minimal unbinding events during the simulation. An average Rg for the CID71496944-PHD2 complex was calculated to be 18.58 Å and is presented in Figure 7C. Finally, the CID11821407-PHD2 complex also showed a stable uniform Rg pattern with minor deviations during the simulation. The Rg pattern of this complex demonstrated alike behavior as its RMSD by reporting a lower Rg value. As given in Figure 7D, an average Rg for this complex was calculated to be 18.44 Å. Overall, the current findings demonstrated minimal unbinding events and preserved the protein packing during the simulation to produce the desired long-term pharmacological effect through the interacting molecules in the binding cavity. This pharmacologically stable behavior prioritized these molecules for the desired clinical therapeutic effects.

### 3.5 Residues flexibility analysis as RMSF

**FIGURE 8:**
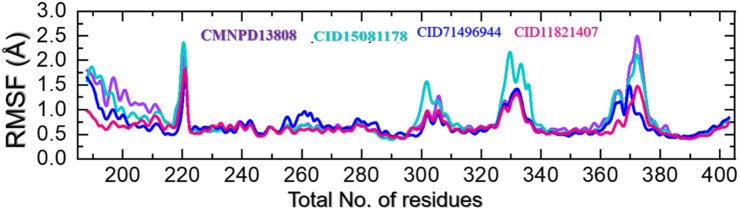
Residue’s flexibility analysis of the CMNPD13808-PHD2, CID15081178-PHD2, CID71496944-PHD2, and CID11821407-PHD2 complexes in a solvated model.

Protein residue flexibility plays a decisive role in the general behavior of a protein and can be affected by numerous elements, including the amino acid sequence, the residues interaction network, and the environment in which the protein is located. In molecular simulations, the thermal motion of atoms in a protein structure is calculated as root mean square fluctuations (RMSF), which describe the deviation of a residue from its average position. RMSF can provide comprehension of the structural changes and the functionality of a protein. The flexibility index can be used to determine the protein-protein interactions, molecular simulation, bio-catalytic mechanism, and mutational impacts and to design new drugs that target specific residues. This approach can also be used to understand the underlying mechanisms that direct the stability and dynamics of proteins (Grottesi et al., 2020). Therefore, RMSF was used here to gain insights into the highly flexible and rigid regions of these complexes. As given in Figure 8, all the complexes demonstrated alike behavior with mostly minimal fluctuations except in the regions 215–224, 298–315, 325–338, and 368–380 which correspond to the loops regions or secondary structural transition elements. These variations in the internal residues’ flexibility show the differential effects of these ligands upon the binding with the key active site residues and produce different levels of inhibitory potential. These results hold promising hopes for the development of effective treatments for diseases associated with HIF-PHD2.

### 3.6 Hydrogen bonding and half-life estimation

**FIGURE 9:**
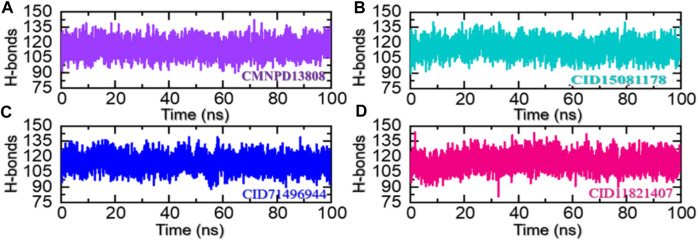
Hydrogen bonding analysis of the ligand-bound protein complexes in the solvated model. **(A)** The H-bonds graph for CMNPD13808-PHD2. **(B)** The H-bonds graph for CID15081178 -PHD2. **(C)** The H-bonds graph for CID71496944-PHD2. **(D)** The H-bonds graph for CID11821407-PHD2.

Hydrogen bonding helps to stabilize the structure of proteins and other biological molecules interacting with the active site residues. It is an important factor in determining the stability of the bound ligand and can have a significant impact on the conformation and function of the protein. The prominence of hydrogen bonding in protein-ligand complexes lies in its ability to strongly and specifically bind ligands to the targeted residues of a protein, which can have substantial pharmacological potential. Understanding and predicting the hydrogen bonding interactions between protein and ligand is, therefore, an important step in the development of new drugs and therapies (Khan et al., 2022b). Hence, we also calculated the total number of hydrogen bonds in each trajectory of the simulation. As given in Figures 9A–D, the average number of hydrogen bonds in the CMNPD13808-PHD2 complex was calculated to be 128, in the CID15081178-PHD2 complex the average number of hydrogen bonds was calculated to be 126, in the CID71496944-PHD2 complex the average number of hydrogen bonds was also calculated to be 126, and, finally, in the CID11821407-PHD2 complex the average number of hydrogen bonds was calculated to be 124.

In molecular dynamics simulations, the half-life of hydrogen bonding refers to the amount of time that a hydrogen bond remains intact during a simulation. Monitoring the changes in the half-life of hydrogen bonds during a simulation can provide information regarding the specific interactions with other molecules (Khan et al., 2022a). Overall, the half-life of hydrogen bonding is an important aspect and provides valuable information regarding the pharmacological potential of a molecule. Herein, we also calculated the half-life of the hydrogen bond for each complex from the simulation trajectory. The hydrogen bond by Asp254 remained consistent in the CMNPD13808-PHD2 complex in 28% of the trajectory which makes 1,400 frames, in the CID15081178\-PHD2 complex in 15% of the trajectory which makes 750 frames, in the CID71496944-PHD2 complex in 31% of the trajectory which makes 1,550 frames, and in the CID11821407-PHD2 complex in 25% of the trajectory which makes 1,250 frames. The hydrogen bond by Tyr303 remained consistent in the CMNPD13808-PHD2 complex in 37% of the total trajectory which makes 1850 frames, in the CID15081178-PHD2 complex in 39% of the total trajectory which makes 1950 frames, in the CID71496944-PHD2 complex in 44% of the total trajectory which makes 2,200 frames, and in the CID11821407-PHD2 complex in 29% of the total trajectory which makes 1,540 frames. Moreover, the hydrogen bond by Arg322 remained consistent in the CMNPD13808-PHD2 complex in 54% of the total trajectory which makes 2,700 frames, in the CID15081178-PHD2 complex in 48% of the trajectory which makes 2,400 frames, in the CID71496944-PHD2 complex in 34% of the total trajectory which makes 1700 frames, and in the CID11821407-PHD2 complex in 43% of the total trajectory which makes 2,150 frames. The hydrogen bond by Tyr303 remained consistent in the CMNPD13808-PHD2 complex in 37% of the total trajectory which makes 1850 frames, in the CID15081178-PHD2 complex in 39% of the total trajectory which makes 1950 frames, in the CID71496944-PHD2 complex in 44% of the total trajectory which makes 2,200 frames, and in the CID11821407-PHD2 complex in 29% of the total trajectory which makes 1,540 frames. Furthermore, the half-life of the Arg383 hydrogen bond in the CMNPD13808-PHD2 complex was reported to be sustained in 73% of the total trajectory which makes 1850 frames, in the CID15081178-PHD2 complex in 78% of the total trajectory which makes 1950 frames, in the CID71496944-PHD2 complex in 69% of the total trajectory which makes 2,200 frames, and in the CID11821407-PHD2 complex in 76% of the total trajectory which makes 1,540 frames. Finally, for Thr387, the half-life in the CMNPD13808-PHD2 complex was reported to be 21% of the total trajectory which makes 1,050 frames, in the CID15081178-PHD2 complex it was 25% of the total trajectory which makes 1,250 frames, in the CID71496944-PHD2 complex it was 36% of the total trajectory which makes 1800 frames, and in the CID11821407-PHD2 complex it was 39% of the total trajectory which makes 1950 frames. Overall, the results show that the essential interaction remained consistent during the simulation for most of the time except Thr387 which is least important for inhibition. In particular, Arg383, which has been reported to play an important role in HIF-PHD2 functionally, was robustly blocked by these molecules with stable hydrogen bonding during the simulation. This shows the potential of these molecules to be tested and used as clinical candidates for the treatment of various diseases. The information regarding the half-life of hydrogen bonds with each active site residue is given in Table 2.

**TABLE 2:** Hydrogen bonding information of the essential active site residues. The table shows the percentage of consistency of a particular hydrogen bond with the calculated frames during the simulation.

Complexes names	Asp254		Tyr303		Arg322		Arg383		Thr387	
	% of trajectory	No. of frames	% of trajectory	No. of frames	% of trajectory	No. of frames	% of trajectory	No. of frames	% of trajectory	No. of frames
CMNPD13808	28	1,400	37	1,850	54	2,700	73	3,650	21	1,050
CID15081178	15	750	39	1,950	48	2,400	78	3,900	25	1,250
CID71496944	31	1,550	44	2,200	34	1,700	69	3,450	36	1,800
CID11821407	25	1,250	29	1,540	43	2,150	76	3,800	39	1,950

### 3.7 Binding free energy of the top hits

Binding free energy, i.e., MM/GBSA (Molecular Mechanics/Generalized Born Solvent Accessible), is a quantification of the energy necessary to bind two or more molecules into a specific binding configuration, and it is a key factor in comprehending the protein-ligand association. By taking into account both the energy required to bring the ligand and protein into a specific binding configuration and the changes in solvent-accessible surface area that occur during binding, the MM/GBSA technique offers a more accurate estimate of binding free energy. It also re-evaluates the docking conformation to determine the accuracy of the binding. In order to create novel drugs, binding free energy calculations are crucial since they offer a quantitative assessment of the potency of a particular binding interaction (Sun et al., 2014). Therefore, in the current study, we also calculated the binding free energy of each complex using the simulation trajectories. The van der Waals forces for these complexes were calculated as −57.41 kcal/mol for CMNPD13808-PHD2, −48.22 kcal/mol for CID15081178-PHD2, −53.89 kcal/mol for CID71496944-PHD2, and −46.02 kcal/mol for CID71496944-PHD2. The electrostatic energy for these complexes was calculated as −16.57 kcal/mol for CMNPD13808-PHD2, −17.11 kcal/mol for CID15081178-PHD2, −9.32 kcal/mol for CID71496944-PHD2, and −14.55 kcal/mol for CID71496944-PHD2. The EGB for these complexes was calculated as 14.22 kcal/mol for CMNPD13808-PHD2, 10.15 kcal/mol for CID15081178-PHD2, 11.21 kcal/mol for CID71496944-PHD2, and 13.78 kcal/mol for CID71496944-PHD2. The ESURF for these complexes were calculated as −13.15 kcal/mol for CMNPD13808-PHD2, -10.37 kcal/mol for CID15081178-PHD2, −16.47 kcal/mol for CID71496944-PHD2, and −15.27 kcal/mol for CID71496944-PHD2 (Table 3). The DELTA G gas for these complexes was calculated as −46.97 kcal/mol for CMNPD13808-PHD2,−43.25 kcal/mo for CID15081178-PHD2 l, −44.17 kcal/mol for CID71496944-PHD2, −42.36 kcal/mol for CID71496944-PHD2. The DELTA G solvated for these complexes was calculated as 12.45 kcal/mol for CMNPD13808-PHD2, 11.25 kcal/mol for CID15081178-PHD2, 9.98 kcal/mol for CID71496944-PHD2, and 10.63 kcal/mol for CID71496944-PHD2. Finally, the total binding free energy for each complex was calculated as −72.91 kcal/mol for CMNPD13808-PHD2, −65.55 kcal/mol for CID15081178-PHD2, -68.47 kcal/mol for CID71496944-PHD2, and −62.06 kcal/mol for CID71496944-PHD2. These drugs possess relatively good activity compared with previously reported drugs and therefore are the best choice for clinical testing (Siddiq et al., 2009; Wu et al., 2017). Consequently, these four hits may inhibit PHD2 more strongly and need further *in vitro* and *in vivo* validation for possible usage as potential drugs against HIF-PHD2-associated diseases.

**TABLE 3:** Total binding free energy for each complex using the simulation trajectories.

Parameters	CMNPD13808	CID15081178	CID71496944	CID11821407
VDWAALS	−57.41	−48.22	−53.89	−46.02
EEL	−16.57	−17.11	−9.32	−14.55
EGB	14.22	10.15	11.21	13.78
ESURF	−13.15	−10.37	−16.47	−15.27
DELTA G gas	−46.97	−43.25	−44.17	−42.36
DELTA G solv	12.45	11.25	9.98	10.63
DELTA TOTAL	−72.91	−65.55	−68.47	−62.06

## 4 Conclusion

To overcome safety issues, liver failure, and problems associated with on-/off-targets associated with HIF-PHD2, natural products offer promising alternatives. In the current study, the natural products chemical space was explored which revealed CMNPD13808, CID15081178, CID71496944, and CID11821407 as promising hits by targeting PHD2 active sites Gln239, Tyr310, His313, Asp315, Tyr329, Arg383, and Trp389 residues. The binding results revealed that the shortlisted hits demonstrated better interaction profiles in contrast to the positive control. Moreover, molecular simulation and binding free energy studies revealed that these drugs possess relatively good activity in comparison with 4HG. The hydrogen bonding results further demonstrated that the essential interactions remained consistent during the simulation for most of the time except for Thr387 which is least important for inhibition. In particular, Arg383, which has been reported to play an important role in HIF-PHD2 functionally, was robustly blocked by these molecules with stable hydrogen bonding during the simulation. To conclude, these hits are potential clinical candidates but further *in silico* experiments such as metadynamics and free energy perturbation analysis, and *in vitro* and *in vivo* validations should be performed to determine the efficacy of these small molecules against HIF-PHD in various diseases.

## Data Availability

The original contributions presented in the study are included in the article/Supplementary Material, further inquiries can be directed to the corresponding author.
